# Antidepressant Effects of *Mallotus oppositifolius* in Acute Murine Models

**DOI:** 10.1155/2014/324063

**Published:** 2014-03-12

**Authors:** Kennedy K. E. Kukuia, Priscilla K. Mante, Eric Woode, Elvis O. Ameyaw, Donatus W. Adongo

**Affiliations:** ^1^Department of Pharmacology, University of Ghana Medical School, College of Health Sciences, University of Ghana, Accra, Ghana; ^2^Department of Pharmacology, Faculty of Pharmacy and Pharmaceutical Sciences, Kwame Nkrumah University of Science and Technology, Kumasi, Ghana; ^3^Department of Biomedical and Forensic Sciences, School of Biological Science, University of Cape Coast, Cape Coast, Ghana

## Abstract

*Objective*. Hydroalcoholic extract of leaves of *Mallotus oppositifolius* (MOE), a plant used for CNS conditions in Ghana, was investigated for acute antidepressant effects in the forced swimming (FST) and tail suspension tests (TST). *Results*. In both FST and TST, MOE (10, 30, and 100 mg kg^−1^) significantly decreased immobility periods and frequencies. A 3-day pretreatment with 200 mg kg^−1^, i.p., para-chlorophenylalanine (PCPA), a tryptophan hydroxylase inhibitor, reversed the decline in immobility and the increase of swimming score induced by MOE in the modified FST. Pretreatment with reserpine alone (1 mg kg^−1^), **α**-methyldopa alone (400 mg kg^−1^, i.p.), or a combination of both drugs failed to reverse the decline in immobility or the increase in swimming score caused by the extract in the modified FST. The extract potentiated the frequency of head twitch responses induced by 5-hydroxytryptamine. Pretreatment with d-serine (600 mg kg^−1^, i.p.), glycine/NMDA agonist, abolished the behavioural effects of MOE while d-cycloserine (2.5 mg kg^−1^, i.p.), a glycine/NMDA partial agonist, potentiated it in both TST and modified FST. *Conclusion*. The extract exhibited antidepressant effects in mice which is mediated by enhancement of serotoninergic neurotransmission and inhibition of glycine/NMDA receptor activation.

## 1. Introduction

Depression is an extremely common pathological complex with psychological, neuroendocrine, and pathological symptoms [1]. It is a leading cause of disability worldwide and has a very significant impact on morbidity, mortality, and health care cost [2–4]. Disconcertion in monoaminergic neurotransmission especially serotonin and noradrenaline neurotransmission is considered the major cause of the observed symptoms of depression. Unfortunately the efficacy of these medications is unsatisfactory and multiple side effects are common [5]. It is estimated that about 40% of patients have conditions refractory to current medications. Furthermore, these drugs require at least 2–4 weeks of administration before producing clinically meaningful improvement in the symptoms [6]. These reasons underpin the need for novel therapeutic agents with less side effects and faster onset of action [7, 8]. Also both preclinical and clinical studies support the role of NMDA receptor antagonists as possible therapeutic agents for depression [5, 9, 10]. For instance ketamine and memantine have demonstrated rapid and profound antidepressant effects clinically [11, 12]. Numerous behavioural studies have further demonstrated that antagonists and partial agonists at the glycine coagonist site of the NMDA receptor have antidepressant potentials with less severe side effects [13]. While the search of newer antidepressants continues, renewed interest in medicinal plants, for example,* Mallotus oppositifolius*, for the treatment of many CNS disorders has been on the ascendancy [14–16].

Despite the use of* Mallotus oppositifolius *in treating psychiatric and affective disorders in Ghana, there is no scientific data on its antidepressant effect. Thus the present study investigated the effect of the hydroalcoholic leaf extract of the plant in acute antidepressant models—the forced swim (FST) and tail suspension tests (TST). The effects of the extract on the monoaminergic system (serotonin and noradrenaline) as well as the glycine/NMDA receptor complex were investigated in order to elucidate the possible mechanism(s) of action of the extract.

## 2. Materials

Leaves of the plant* Mallotus oppositifolius* were collected from the wild around the Kwame Nkrumah University of Science and Technology (KNUST), Kumasi, Ghana (6°41′6.4′′N, 1°33′42.8′′W) and authenticated at the Department of Herbal Medicine of the Faculty of Pharmacy and Pharmaceutical Sciences, KNUST, Kumasi, where a voucher specimen (KNUST/FP/035/09) has been deposited. After air-drying for 7 days, the leaves were pulverized with a hammer-mill and the powder extracted by cold maceration using 70% (v/v) ethanol in water over a period of 72 h. The resulting extract was concentrated under moderate temperature (60°C) and pressure to a syrupy mass on a rotary evaporator. The syrupy mass was then dried to a dark brown semisolid mass using water bath and kept in a desiccator till it was ready to be used. The final yield was 9.5% (w/w). This is subsequently referred to as* Mallotus oppositifolius* extract (MOE) or extract.

### 2.1. Animals

Male ICR mice were obtained from and housed at the animal facility of the Department of Pharmacology, KNUST, Kumasi, Ghana. The animals were housed in groups of five in stainless steel cages (34 × 47 × 18 cm) with soft wood shavings as bedding, fed with normal commercial pellet diet (GAFCO, Tema), given water* ad libitum*, and maintained under laboratory conditions. All animals used in these studies were treated in accordance with the Guide for the Care and Use of Laboratory Animals [17] and experiments were approved by the College Ethics Committee.

### 2.2. Chemicals

Fluoxetine hydrochloride (Prozac) was from Eli Lilly and Co., Basingstoke, England. Imipramine hydrochloride (Tofranil) from Mallinckrodt Pharmaceuticals, Ireland. Desipramine hydrochloride, d-serine, d-cycloserine, *α*-methyldopa, reserpine, and para-chlorophenylalanine were purchased from Sigma-Aldrich Inc., St. Louis, MO, USA.

### 2.3. Forced Swimming Test (FST)

The FST was based on that described by Porsolt et al. [18]. Mice were divided into ten groups of five animals each and received the vehicle (water), extract (10, 30, or 100 mg kg^−1^,* p.o*.), or the standard reference drugs fluoxetine (3, 10, or 30 mg kg^−1^,* p.o*.), imipramine (3, 10, or 30 mg kg^−1^,* p.o*.). One hour after oral administration of the test compounds, mice were gently placed individually into transparent cylindrical polyethylene tanks (25 cm high, 10 cm internal diameter) containing water (25 to 28°C) up to a level of 20 cm and left there for 5 min. Each session was recorded by a video camera suspended approximately 100 cm above the cylinders. An observer scored the duration of immobility (when mouse floated upright in the water and made only small movements to keep its head above water), during the last 5 min test, from the videotapes with the aid of the public domain software JWatcher Version 1.0 (University of California, Los Angeles, USA, and Macquarie University, Sydney, Australia, available at http://www.jwatcher.ucla.edu/).

### 2.4. Tail Suspension Test

The TST was carried out as previously described by Steru et al. [19]. Mice were allowed to acclimatize to the room for 3.5–4 h before the test. Groups of ten mice were treated with MOE (10, 30, or 100 mg kg^−1^,* p.o*.), fluoxetine (3, 10, or 30 mg kg^−1^,* p.o*.), and imipramine (3, 10, or 30 mg kg^−1^,* p.o*.) or vehicle. One hour after oral administration of the test compounds, mice were individually suspended by the tail from a horizontal bar (distance from floor = 30 cm) using adhesive tape (distance from tip of tail = 1 cm). Duration of immobility, defined as the absence of all movement except for those required for respiration, was recorded by an observer for 5 min from video recordings of the test as described above for forced swimming test. Mice that climbed up on their tails during the test session were gently pulled down and testing continued. Mice that continued to climb their tails were excluded from the study.

### 2.5. Involvement of Noradrenergic Systems

Mice were pretreated with reserpine and/or *α*-methyldopa (*α*-MD) in order to investigate the possible role of noradrenergic system in the actions of MOE [20]. The doses of *α*-MD and reserpine were chosen on the basis of work done by others [20, 21]. To deplete newly synthesized pools of noradrenaline (NE) and dopamine (DA), mice were treated with a single dose of *α*-MD (400 mg kg^−1^, i.p.) 3.5 hours before behavioural testing. To deplete vesicular pools of NE and DA, mice were treated with a single dose of reserpine (1 mg kg^−1^, s.c.) 24 h before behavioral testing. In an effort to deplete both the vesicular and cytoplasmic pools of NE and DA, mice were pretreated with a combination of reserpine (1 mg kg^−1^, s.c., 24 h before behavioral testing) and *α*-MD (200 mg kg^−1^, i.p., 3.5 hours before behavioral testing), respectively.

### 2.6. Involvement of Serotoninergic Systems

Mice were grouped into groups of five (5) forming ten (20) groups.* p*CPA (200 mg kg^−1^, i.p.) was administered once daily for 3 consecutive days to some of the animals. On the fourth day, group 1 received saline without pretreatment; group 2 received* p*CPA after pretreatment; groups 3 to 5 received MOE (10, 30, and 100 mg kg^−1^) without pretreatment; groups 6 to 8 received MOE (10, 30, and 100 mg kg^−1^,* p.o*.) after pretreatment; groups 9 to 11 received fluoxetine (3, 10, and 30 mg kg^−1^,* p.o*) alone; groups 12 to 14 received fluoxetine (3, 10, and 30 mg kg^−1^,* p.o*.) after pretreatment; groups 15–17 received imipramine (3, 10, and 30 mg kg^−1^,* p.o*.) alone; and finally groups 18–20 received imipramine (3, 10, and 30 mg kg^−1^,* p.o*.) after pretreatment. After the tail suspension sessions, mice were taken through the modified forced swimming test. The modified forced swimming test followed the same procedure described above except that the depth of water was changed to 20 cm. For tail suspension, immobility period was scored, whilst for the modified swimming test mean immobility counts, mean swimming counts and mean climbing counts were scored.

### 2.7. Involvement of Glycine/NMDA Neurotransmission

Mice were divided into 2 groups, A and B. Groups A and B were further subdivided into 6 groups each (*n* = 8). Briefly five groups of mice from group A received d-cycloserine (2.5 mg kg^−1^, i.p.) and 30 min after the first three groups received an oral dose of the extract (10–100 mg kg^−1^) with the last two groups receiving either fluoxetine (10 mg kg^−1^) or desipramine (10 mg kg^−1^, i.p.). The sixth group received only d-cycloserine. Again five groups of mice from group B received d-serine (600 mg kg^−1^) and 30 min after the first three groups received an oral dose of the extract (10–100 mg kg^−1^) with the last two groups receiving either fluoxetine (10 mg kg^−1^,* p.o*.) or desipramine (10 mg kg^−1^, i.p.). The sixth group from group B received only d-serine. The forced swim and tail suspension tests were used as described above to investigate the antidepressant mechanism. Pedalling behaviour was defined as the continuous movement of the paw of the mice without moving the body while curling was defined as the raising of the head of the mouse towards its hind paws.

### 2.8. Statistics

GraphPad Prism for Windows version 4.03 (GraphPad Software, San Diego, CA, USA) was used for all data and statistical analyses. *P* < 0.05 was considered statistically significant. In all the tests, a sample size of ten animals (*n* = 10) were used. The time-course curves were subjected to two-way (treatment × time) repeated measures analysis of variance (ANOVA) with Bonferroni's* post hoc* test. Total immobility time, distance travelled, and time taken to find the hidden platform and change in weight for each treatment were calculated in arbitrary unit as the area under the curve (AUC). Differences in AUCs were analysed by ANOVA followed by Newman Keuls'* post hoc* test.

## 3. Results

### 3.1. Forced Swimming and Tail Suspension Tests

**Figure 1:**
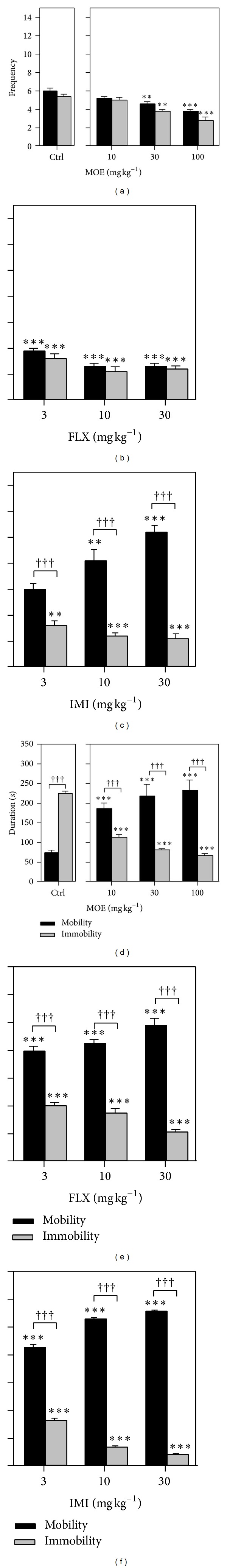
Effects of extract, MOE (10–100 mg kg^−1^), fluoxetine, FLX (3–30 mg kg^−1^), and imipramine, IMI (3–30 mg kg^−1^), treatment on (a, b, and c) the frequency of mobility and immobility and (d, e, and f) duration of mobility and immobility in the FST. Data are presented as group means ± SEM, significantly different from control: ***P < 0.001; **P < 0.01 (one-way ANOVA followed by Newman Keuls' test). ^†††^ *P* < 0.001, comparison between effect and dose (two-way ANOVA followed by Bonferroni's test).

**Figure 2:**
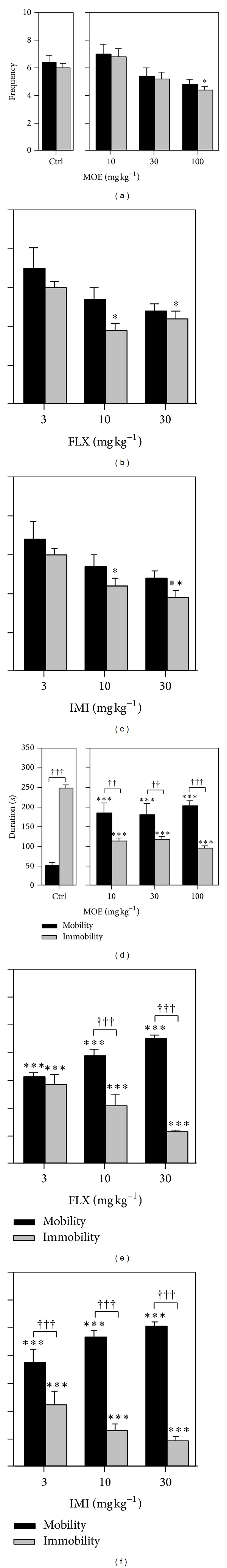
Effect of the extract, MOE (10, 30, and 100 mg kg^−1^), fluoxetine, FLX (3, 10, and 30 mg kg^−1^), and imipramine, IMI (3, 10, and 30 mg kg^−1^) treatment on the (a, b, and c) frequency of mobility and immobility and (d, e, and f) duration of mobility and immobility in the TST. Data are presented as group means ± SEM. ***P < 0.001; **P < 0.01; compared to vehicle-treated group (one-way ANOVA followed by Newman Keuls' test). ^†††^ *P* < 0.001, comparison effect and dose (two-way ANOVA followed by Bonferroni's test).

MOE (10–100 mg kg^−1^,* p.o.*), administered 60 min before the test period, significantly decreased the frequency of immobility (*F*_3,19_ = 21.47, *P* < 0.001) (Figure 1(a)) and immobility periods of mice (*F*_3,19_ = 143.4, *P* < 0.001) (Figure 1(d)) in a dose dependent manner in the FST. In the TST both frequency (*F*_6,54_ = 0.486, *P* = 0.8159) (Figures 2(a), 2(b), and 2(c)) and duration (*F*_6,52_ = 25.57, *P* < 0.001) (Figures 2(d), 2(e), and 2(f)) of immobility decreased, indicating significant antidepressant activity.

### 3.2. Involvement of Noradrenergic Mechanisms

**Figure 3:**
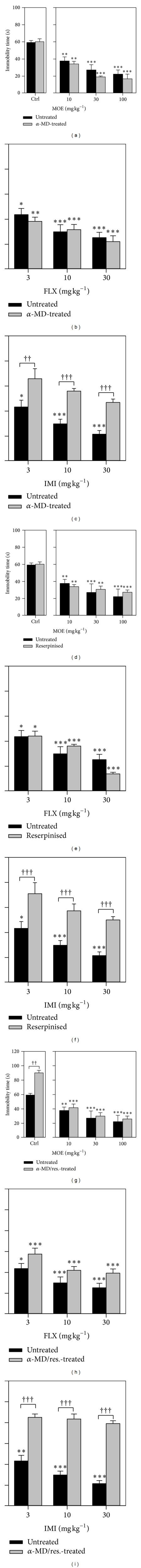
Effects of (a–c) reserpine alone; (d–f) *α*-methyldopa, *α*-MD, alone; or (g–i) both reserpine and *α*-methyldopa on duration of immobility of MOE (10–100 mg kg^−1^,* p.o*.), fluoxetine (3–30 mg kg^−1^,* p.o*.), and imipramine (3–30 mg kg^−1^,* p.o*.) treatment in the FST. Data are presented as mean ± SEM of 5 animals, significantly different from control: **P < 0.01, ***P < 0.001 by one-way ANOVA followed by Newman Keuls' test. ^†^ *P* < 0.05, ^††^ *P* < 0.01, and ^†††^ *P* < 0.001; significant difference between treatment and dose (two-way ANOVA with Bonferroni* post hoc* test).

Pretreatment with reserpine (1 mg kg^−1^, s.c.) alone, *α*-methyldopa (400 mg kg^−1^,* p.o*.) alone, or a concomitant administration of reserpine (1 mg kg^−1^, s.c.) and *α*-methyldopa (200 mg kg^−1^,* p.o*.) did not reverse the decline in immobility caused by the extract, MOE (10–100 mg kg^−1^,* p.o*.), in the forced swim test (FST) (Figures 3(a), 3(d), and 3(g)). Results obtained for fluoxetine-treated groups (FLX) (3–30 mg kg^−1^,* p.o*.) were similar to that of the extract-treated groups (Figures 3(b), 3(e), and 3(h)). In contrast, the antidepressant effect of imipramine (IMI) was reversed by either reserpine alone, *α*-methyldopa alone, or a concomitant administration of reserpine and *α*-methyldopa (Figures 3(c), 3(f), and 3(i)).

### 3.3. Involvement of Serotoninergic Mechanism

**Figure 4:**
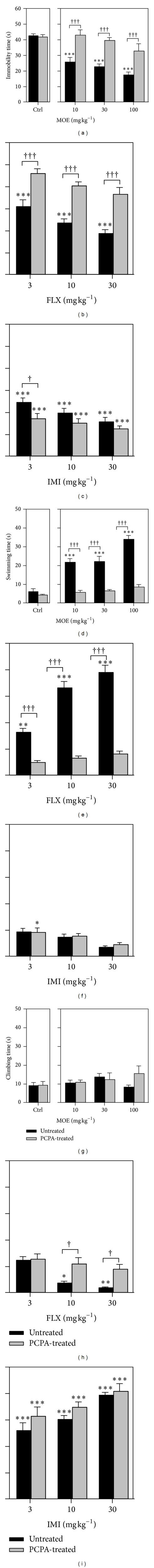
Effects of* p*CPA (200 mg kg^−1^) pretreatment on the (a–c) mean immobility counts, (d–f) swimming counts, and (g–i) climbing counts of oral doses of extract, MOE (10–100 mg kg^−1^), fluoxetine, FLX (3–30 mg kg^−1^), and IMI (3–30 mg kg^−1^) treated groups in the FST. Data are presented as mean ± SEM, significantly different from control: **P < 0.01, ***P < 0.001 by Newman Keuls' test. ^†††^ *P* < 0.001 (two-way ANOVA followed by Bonferroni's post test, comparison between drug treatment and dose).

Pretreatment of mice with* p*CPA (200 mg kg^−1^) abolished the antidepressant effect of MOE (10–100 mg kg^−1^,* p.o.*), and fluoxetine, FLX (3–30 mg kg^−1^,* p.o.*), but not imipramine, IMI (3–30 mg kg^−1^,* p.o*.), in the FST. The mean counts for immobility (*F*_7,32_ = 14.63; *P* < 0.0001) (Figure 4(a)), swimming (*F*_7,32_ = 44.74; *P* < 0.0001) (Figure 4(d)), and climbing (*F*_7,32_ = 1.121; *P* = 0.3742) (Figure 4(g)) in the extract-treated group after* p*CPA pretreatment did not show any difference when compared with the control. Similar results as above were observed for FLX-treated groups but not imipramine (Figures 4(b)-4(c), 4(e)-4(f), and 4(h)-4(i)).

**Figure 5:**
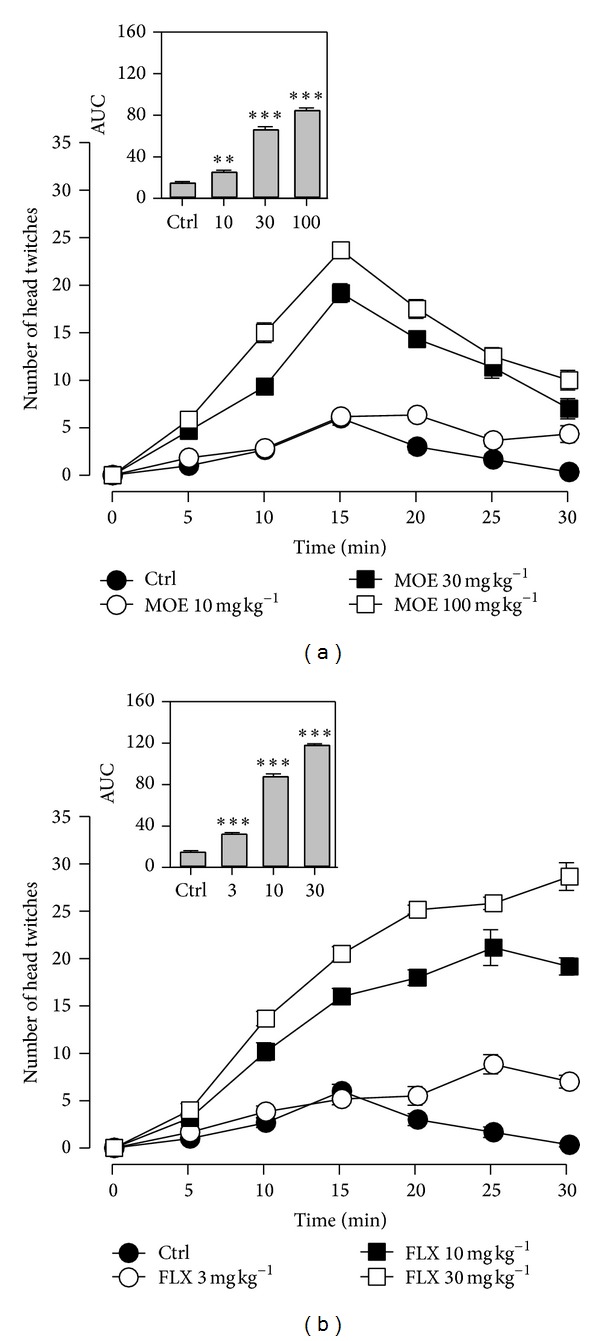
Effect of MOE (10–100 mg kg^−1^,* p.o*.) and fluoxetine (3–30 mg kg^−1^,* p.o*.) on the time course curve of head twitch response test and their corresponding AUCs (a and b) in the same test represented as bar graphs. Data was presented as mean ± SEM. (*n* = 6); ***P < 0.001; **P < 0.01; compared to vehicle-treated group (one-way ANOVA followed by Newman Keuls' test).

In an attempt to investigate the possible involvement of 5-HT_2A_ receptor activation in the antidepressant action of the extract, mice were given 5-hydroxytryptophan after extract pretreatment to induced head twitch responses. It was observed from the time course curve that the extract as well as fluoxetine increased the head twitch responses significantly for the period of 30 minutes (Figures 5(a) and 5(c)). Response peaked after 15 minutes. One-way ANOVA followed by Newman Keuls' test of the areas under the curve (AUCs) showed a dose dependent increase in the head twitch response for both extract and fluoxetine (Figures 5(b) and 5(d)).

### 3.4. Involvement of Glycine/NMDA Receptor Complex

**Figure 6:**
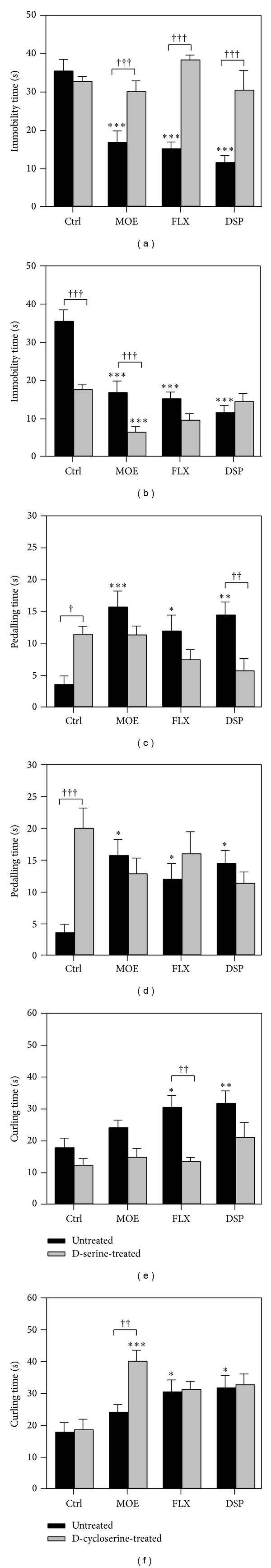
Effects of d-serine (DS) or d-cycloserine (DCS) pretreatment on (a, b) mean immobility count, (c, d) pedalling count, and (e, f) curling count of extract, MOE (100 mg kg^−1^), fluoxetine, FLX (10 mg kg^−1^), and desipramine, DSP (10 mg kg^−1^), treatment in the tail suspension test (TST). Data are presented as Means ± SEM, significantly different from control: **P < 0.01, ***P < 0.001 by Newman Keul's test. Significant difference between treatments: ^†^ *P* < 0.05, ^††^ *P* < 0.01, and ^†††^ *P* < 0.001.

In the TST, MOE (100 mg kg^−1^,* p.o*.), fluoxetine, FLX (10 mg kg^−1^,* p.o.*), and desipramine, DSP (10 mg kg^−1^, i.p.), exhibited significant antidepressant effect by decreasing mean immobility score which was reversed by d-serine, DS (600 mg kg^−1^, i.p.), pretreatment (Figure 6(a)). Pretreatment with d-cycloserine, DCS (2.5 mg kg^−1^, i.p.), potentiated the effect of MOE and FLX (but not DSP) by further decreasing mean immobility score (Figure 6(b)). MOE alone did not affect curling score and this was not changed by DS pretreatment (Figure 6(e)). FLX and DSP alone caused slight increase in the curling score which was reversed by DS. Pretreatment with DCS significantly increased curling score of MOE but caused only a modest increase in both FLX and DSP treated groups (Figure 6(f)). MOE, DS, DCS, FLX, and DSP alone increased swinging score. DS pretreatment partially inhibited swinging behaviour by MOE but totally in FLX and DSP treated groups (Figure 6(c)). DCS pretreatment also inhibited swinging behaviour by MOE and DSP but not FLX (Figure 6(d)).

**Figure 7:**
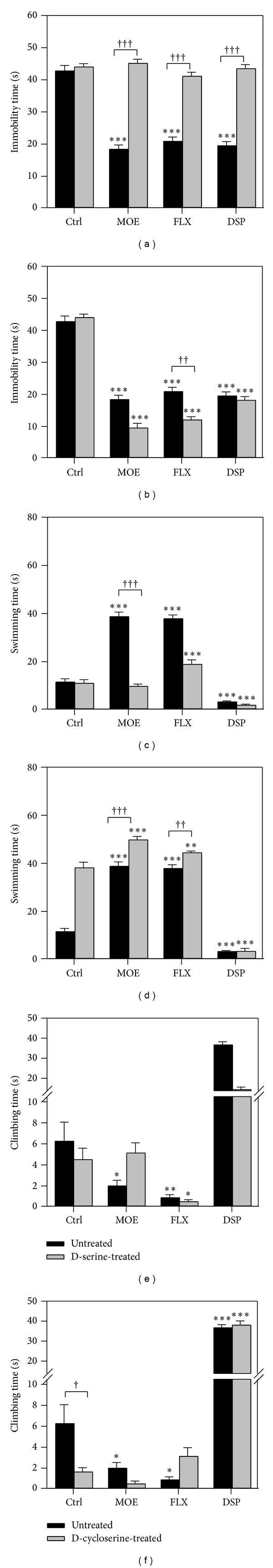
Effects of d-serine (DS) or d-cycloserine (DCS) pretreatment on (a, b) mean immobility count, (c, d) swimming count, and (e, f) climbing count of extract, MOE (100 mg kg^−1^), fluoxetine, FLX (10 mg kg^−1^), and desipramine, DSP (10 mg kg^−1^), treatment in the forced swimming test (FST). Data are presented as means ± SEM, significantly different from control: **P < 0.01, ***P < 0.001 by Newman Keul's test. Significant difference between treatments: ^†^ *P* < 0.05, ^††^ *P* < 0.01, and ^†††^ *P* < 0.001.

In the forced swim test, MOE, FLX, and DSP decreased immobility score and this was reversed by DS pretreatment (Figure 7(a)). Pretreatment with DCS potentiated the effect of MOE and FLX (but not DSP) by further decreasing immobility score (Figure 7(b)). MOE and FLX, unlike DSP, increased swimming behaviour which was inhibited by DS but increased by DCS pretreatment (Figures 7(c) and 7(d)). Climbing scores were decreased by both MOE and FLX and this was not affected by both DS and DCS pretreatment (Figures 7(e) and 7(f)). DSP, on the contrary, increased climbing score which was unaffected by DCS pretreatment but decreased by DS.

## 4. Discussion

Results of the study demonstrated that MOE has significant antidepressant effect in both the forced swimming and the tail suspension tests. In both animal models, percentage immobility and frequency of immobility were decreased by extract treatment. Reduction in immobility has been used as the primary index for antidepressant effect of test substances in these models—almost all antidepressants in clinical use induce a decrease in immobility in rodents whilst other drugs fail to give the same response [22, 23].

Preclinical and clinical studies suggest that depletion of a monoamine implicated in depression pathophysiology may abolish the antidepressant effect of a substance if the substance depends on that particular monoamine for its antidepressant effect [20, 24]. Hence when 5-HT was depleted by pretreating mice for 3 days with the tryptophan hydroxylase inhibitor para-chlorophenylalanine (*p*CPA), the effects of drugs that act by enhancing 5-HT neurotransmission were abolished [25, 26] while those that act on noradrenergic pathways were not affected [26–28]. The inhibition of the antidepressant effect of MOE by* p*CPA in both TST and FST suggests that its antidepressant effect is dependent on enhancement of serotoninergic neurotransmission. The lack of antidepressant effect of fluoxetine in* p*CPA-treated mice is consistent with the hypothesis that fluoxetine elicits its acute behavioural effects by increasing extracellular 5-HT after blockade of the serotonin transporter [29]. The extract and fluoxetine increased swimming score which was reversed with* p*CPA pretreatment, further supporting their action on the serotoninergic system [27, 30]. Both MOE and fluoxetine did not affect mean climbing score, suggesting that their behavioural effect may not depend on noradrenergic pathways.

Further evidence suggesting that the extract enhances 5-HT neurotransmission was derived from its ability to increase head twitch responses induced by 5-HTP. The head twitch response (HTR) in rodents induced by 5-hydroxytryptophan (5-HTP), a precursor of 5-HT [31], is considered as a specific behavioural model for the activation of serotoninergic neuron specifically 5-HT_2A_ receptors [32]. Thus it can be inferred that MOE may be acting via direct or indirect activation of 5-HT_2A_ receptors. Fluoxetine also increased the frequency of HTRs. This result is consistent with a number of studies where fluoxetine elicited similar responses [33, 34].

The present experiments also examined the role of noradrenaline and dopamine in the acute behavioural effects of the extract in the modified FST by using drugs that interfere with their neurotransmitter synthesis or release. Depletion with *α*-methyldopa, an L-aromatic amino acid decarboxylase inhibitor that inhibits the biosynthesis of catecholamines and 5-HT [35], failed to attenuate the behavioural effects of MOE and fluoxetine while that of imipramine was abolished. This suggests that MOE may not be affected by the biosynthesis of noradrenaline or dopamine. Moreover, when vesicular pools were depleted by reserpine, the decrease in immobility elicited by MOE or fluoxetine was not affected. Here again the effect of imipramine was attenuated. Reserpine is an irreversible inhibitor of the vesicular monoamine transporter 2 (VMAT-2) which is located primarily within the CNS and is responsible for transporting monoamines from the cytoplasm into secretory vesicles [36, 37]. Treatment with reserpine therefore leads to depletion of vesicular monoamine stores—both serotonin and noradrenaline [38] suggesting both serotonin and noradrenaline might be important in the antidepressant effects of imipramine. The inability of reserpine pretreatment to reverse the antidepressant effects of the extract and fluoxetine, however, seems to suggest that reserpine does not affect vesicular storage of 5-HT to the same extent as that of noradrenaline. In fact this assertion is consistent with the results obtained by O'Leary et al. and Woode et al. [20, 39]—the former demonstrating that reserpine at the dose used produced a 93 and 95% depletion of cortical noradrenaline and dopamine content, respectively, and a 78% depletion of 5-HT. To inhibit synthesis as well as deplete vesicular pools of noradrenaline and dopamine, mice were pretreated with both reserpine and *α*-methyldopa. Results were similar to effects observed when mice were treated with reserpine alone. The work published by O'Leary et al. [20] indicated that, when reserpine was combined with *α*-methyl para-tyrosine AMPT (NE and DA biosynthetic inhibitor), a depletion of cortical DA (95%), NE (97%), and 5-HT (78%) was observed. The combination had only a modest effect on NE and DA but failed to affect 5-HT. Though *α*-methyldopa was used instead of the AMPT used by O'Leary and colleagues, the results from the combined effect of reserpine and *α*-methyldopa did not differ significantly from when reserpine was used alone. A more recent publication by Woode et al. [39] used *α*-methyldopa and similar results was observed. These results demonstrate that the antidepressant effect of MOE may not be dependent on noradrenergic neurotransmission.

Both clinical and preclinical studies support the antidepressant activity of antagonists on functional glycine/NMDA receptor complex. These compounds are thought to have lower side effect profiles compared to the competitive and noncompetitive NMDA antagonists. The effect of MOE on glutamatergic neurotransmission was assessed by pretreating mice with d-serine (DS), a full agonist on glycine/NMDA receptors, or d-cycloserine (DCS), a partial agonist on these receptors. In both the TST and FST, DS reversed the decline in immobility by MOE and fluoxetine but DCS pretreatment potentiated the decline, demonstrating that MOE may be acting as an antagonist on the glycine/NMDA receptor complex. In contrast, the decrease in immobility of desipramine was reversed by DS but DCS had no effect on it. This suggests that the antidepressant effect of both serotonin and noradrenaline-based compounds depends on the inhibition of the glycine/NMDA receptor complex but the enhancement of antidepressant activity depends on the serotoninergic pathway and not the noradrenaline pathway [40]. This explains why the antidepressant effect of the extract, fluoxetine, and desipramine was abolished by d-serine but only the effect of the extract and fluoxetine (which act via serotoninergic pathway) was potentiated by d-cycloserine. Moreover, MOE increased curling score in the TST slightly (suggestive of opioidergic activity) though not significant. Pretreatment with DCS significantly increased curling score of MOE but did not affect both FLX and DSP treated groups. DS pretreatment partially inhibited pedaling behaviour by MOE but totally in FLX and DSP treated groups. DCS pretreatment also inhibited pedaling behaviour by MOE and DSP but not FLX. According to Berrocoso et al. [41], opioids decrease immobility score while increasing curling behaviour in mice. This suggests that the extract on its own may have little effect on opioidergic activity but may interact with opioid receptors when combined with DCS. In the FST, MOE and FLX unlike DSP increased swimming behaviour which is sensitive to selective serotonin reuptake inhibitors (SSRIs) and 5HT agonists [22, 42]. This behavior exhibited by MOE and fluoxetine-treated mice was inhibited by DS but increased by DCS pretreatment further supporting the theory that antidepressant effect of serotonin based drugs depends on the inhibition of the glycine/NMDA receptor complex and the enhancement of antidepressant activity depends on the serotoninergic pathway [40]. Climbing scores were decreased by both MOE and FLX and this was not affected by both DS and DCS pretreatment. This confirms their lack of noradrenergic activity. DSP, a selective noradrenergic reuptake inhibitor, on the contrary, increased climbing score which was unaffected by DCS pretreatment but decreased by DS. It is worth mentioning that the extract, fluoxetine, desipramine alone, or their combination with either d-serine or d-cycloserine did not impair motor coordination. Thus the behavioural effect observed can be attributed to drug treatment alone.

## 5. Conclusion

The present study shows that* Mallotus oppositifolius* has antidepressant-like effect in mice and this may be mediated via enhancement of serotoninergic neurotransmission and inhibition of glycine/NMDA receptor complex activation. The antidepressant effect of the extract may be devoid of noradrenergic mechanisms.
